# Comprehensive Analysis of Transcriptome Sequencing Data in the Lung Tissues of COPD Subjects

**DOI:** 10.1155/2015/206937

**Published:** 2015-03-05

**Authors:** Woo Jin Kim, Jae Hyun Lim, Jae Seung Lee, Sang-Do Lee, Ju Han Kim, Yeon-Mok Oh

**Affiliations:** ^1^Department of Internal Medicine and Environmental Health Center, Kangwon National University Hospital, School of Medicine, Kangwon National University, Chuncheon 200-722, Republic of Korea; ^2^Seoul National University Biomedical Informatics and Systems Biomedical Informatics Research Center, Division of Biomedical Informatics, Seoul National University College of Medicine, Seoul 110-799, Republic of Korea; ^3^Department of Pulmonary and Critical Care Medicine, and Clinical Research Center for Chronic Obstructive Airway Diseases, Asan Medical Center, University of Ulsan College of Medicine, Seoul 138-736, Republic of Korea

## Abstract

*Background and Objectives.* Chronic obstructive pulmonary disease (COPD) is a complex disease characterized by airflow limitation. Although airway inflammation and oxidative stress are known to be important in the pathogenesis of COPD, the mechanism underlying airflow obstruction is not fully understood. Gene expression profiling of lung tissue was performed to define the molecular pathways that are dysregulated in COPD.* Methods.* RNA was isolated from lung tissues obtained from 98 subjects with COPD and 91 control subjects with normal spirometry. The RNA samples were processed with RNA-seq using the HiSeq 2000 system. Genes expressed differentially between the two groups were identified using Student's* t*-test.* Results.* After filtering for genes with zero counts and noncoding genes, 16,676 genes were evaluated. A total of 2312 genes were differentially expressed between the lung tissues of COPD and control subjects (false discovery rate corrected *q* < 0.01). The expression of genes related to oxidative phosphorylation and protein catabolism was reduced and genes related to chromatin modification were dysregulated in lung tissues of COPD subjects.* Conclusions*. Oxidative phosphorylation, protein degradation, and chromatin modification were the most dysregulated pathways in the lung tissues of COPD subjects. These findings may have clinical and mechanistic implications in COPD.

## 1. Introduction

Chronic obstructive pulmonary disease (COPD) is characterized by chronic airflow limitation that is not fully reversible and is highly prevalent worldwide [1]. Although cigarette smoking is a major risk factor for COPD, the mechanism by which inhaled smoke contributes to airflow obstruction is not fully understood. Current theories for this include persistent airway inflammation that is modified by oxidative stress, excess proteinases, autoimmunity, and apoptosis of alveolar cells [2].

Gene expression studies of diseased lungs can generate high-throughput results to shed light on the molecular processes underlying COPD pathogenesis. Whole-genome expression of COPD in humans has been studied using airway epithelium and resected lung tissue in several groups [3–9]. These studies mostly used microarrays to profile gene expression patterns in COPD. Next-generation sequencing technology was recently applied to transcriptomics. RNA-seq technology provides read counts of RNA fragments in each gene [10]. Background and cross-hybridization are not issues in RNA-seq and the technology can quantify both lowly and highly abundant transcripts [11]. In addition, this method can provide information about splice variants, allele-specific expression, and fusion transcripts [12]. RNA-seq data of airways was recently published [13, 14]; however, the number of subjects in these studies was relatively small.

We performed gene expression profiling using RNA-seq of lung tissues resected from large numbers of subjects with COPD or with control subjects to better understand the molecular mechanisms responsible for the pathogenesis of COPD.

## 2. Methods

### 2.1. Study Populations

Subjects were patients who required resection for lung cancer and who were registered in the Asan Biobank from January 2008 to November 2011. The inclusion criteria were a postbronchodilator FEV_1_/FVC ratio (ratio of forced expiratory volume in the first second to forced vital capacity) of less than 0.7 for the COPD group and normal spirometry for the control group in accordance with American Thoracic Society/European Respiratory Society criteria [15]. This study was approved by the institutional review board of Asan Medical Center (2011-0711) and written informed consent was obtained from all patients.

### 2.2. RNA Preparation and Sequencing

Total RNA was isolated from apparently normal fresh frozen lung tissue that was remote from the lung cancer. RNA integrity was assessed using an Agilent Bioanalyzer and RNA purity was assessed using a NanoDrop spectrophotometer.

One *μ*g of total RNA was used to generate cDNA libraries using the TruSeq RNA library kit. The protocol consisted of poly A-selected RNA extraction, RNA fragmentation, reverse transcription using random hexamer primers, and 100 bp paired-end sequencing using the Illumina HiSeq 2000 system. All data have been deposited in the NCBI Gene Expression Omnibus (GEO) public repository and can be accessed through the accession number GSE57148.

### 2.3. Quality Control and Data Management

For quality control, read quality was verified using FastQC and read alignment was verified using Picard. Differential gene expression (DEG) analysis was performed using TopHat and Cufflinks software [16]. To estimate expression levels, the RNA-seq reads were mapped to the human genome using TopHat (version 1.4.1) [17] and quantified using Cufflinks software 2.0.0 [18]. Cufflinks software was run with the UCSC hg19 human genome and transcriptome references. The numbers of isoform and gene transcripts were calculated and the relative abundance of transcripts was measured in fragments per kilobase of exon per million fragments mapped (FPKM).

**Figure 1:**
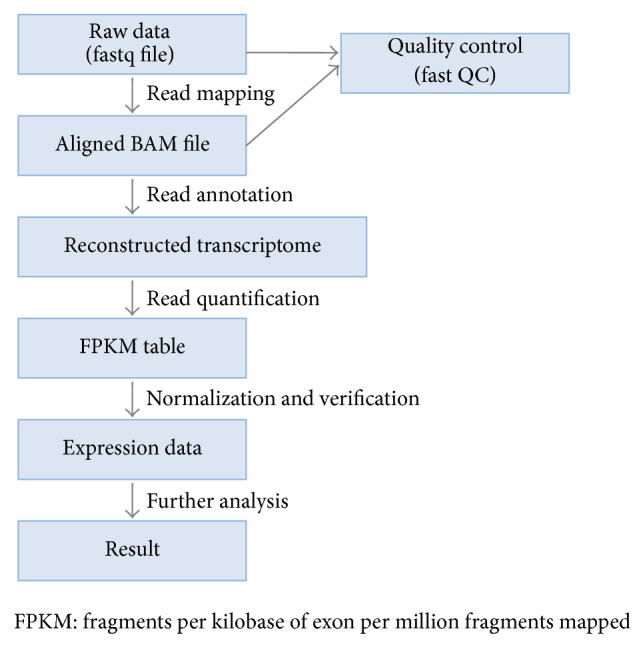
Schematic overview of the transcript analysis of RNA-seq experiment. Briefly, we used TopHat to align raw fastq files and used Cufflinks to read annotation and quantification. FastQC was used to check read quality.

Expression levels were extracted as a FPKM value for each gene of each sample using Cufflinks software. Genes with FPKM values of 0 across all samples were excluded. Filtered data were subject to upper quantile normalization. Statistical significance was determined using Student's *t*-test. The false discovery rate (FDR) was controlled by adjusting *P* values using the Benjamini-Hochberg algorithm. The analysis steps used are summarized in Figure 1.

To investigate whether DEGs are related to clinical phenotypes, we performed a linear regression analysis for 5 clinical phenotypes with respect to its gene expression. We considered each clinical phenotype as the responder for regression and each gene expression as the predictor.

### 2.4. Quantitative Real-Time PCR (qRT-PCR)

Several genes whose expression level was found to be related to COPD status by RNA-seq were validated using TaqMan real-time PCR. The results were normalized to GAPDH Ct values. Primer sequences for the genes of interest are given in Table 2.

### 2.5. Pathway Analysis

Functional enrichment analysis was performed using gene set enrichment analysis (version 2.0.8), which combines information from previously defined gene sets obtained from the Molecular Signature Database (version 3.1). Biological gene functional annotation analysis was performed using DAVID (version 6.7) with a list of DEGs. BioLattice (version 1.1) was used to annotate coexpressed gene groups to GO biological process terms and visualize their relations [19].

### 2.6. Differential Alternative Splicing

To detect differential alternative splicing between the two groups, subjects from each group were evaluated using a multivariate Bayesian algorithm called “multivariate analysis of transcript splicing” [20]. Differential alternative splicing, including exon skipping, mutually exclusive exons, alternative 5′ or 3′ splice site usage, and intron retention, was investigated. Exon usage of* VIM* between two groups was visualized using DEXSeq [21].

### 2.7. Connectivity Map

A connectivity map [22] was used to identify potential drugs that might reverse the gene expression pattern associated with the pathogenesis of early COPD. The connectivity map is a collection of genome-wide transcriptional expression data from cultured human cells treated with bioactive small molecules. The basic assumption of the connectivity map is that transcriptional perturbation can occur or be treated by certain drugs that intrigue similar changes.

## 3. Results

### 3.1. Demographic Characteristics

The demographic characteristics of 98 subjects with COPD and 91 control subjects are shown in Table 1. All subjects were male and the mean age and the mean pack years of cigarette smoking history were higher in the COPD group than in the control group. As expected, pulmonary function was significantly lower in the COPD group than in the control subjects. Most of COPD subjects were in early stage. In COPD group, 28 subjects took inhaled corticosteroid, 40 subjects took tiotropium, and 22 subjects took short-acting beta-agonist. None in control group took bronchodilator.

### 3.2. Quality Control and DEGs

**Figure 2:**
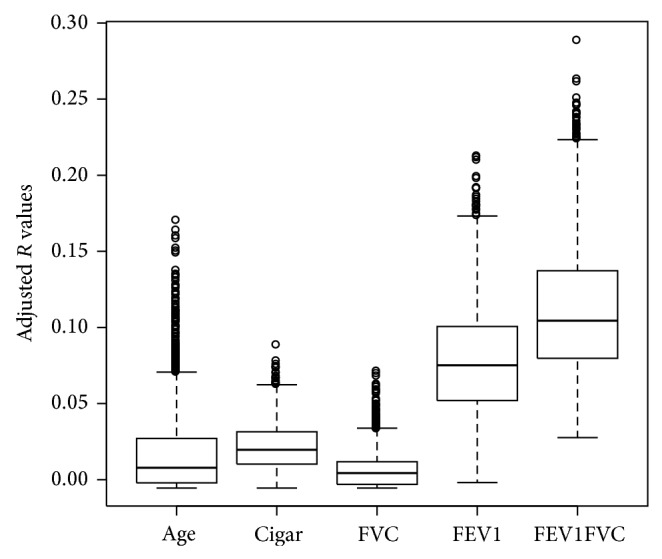
Regression analysis of the differentially expressed genes with clinical phenotypes. Genes were more associated with FEV_1_ (forced expiratory volume in 1 second) and FEV_1_/FVC ratio (ratio of forced expiratory volume in 1 second to forced vital capacity) than with age and smoking history.

The total number of reads produced from each sample was 38,742,474 ± 7,332,014 reads (mean ± standard deviation). The difference in the number of reads between COPD samples and control samples was not statistically significant. After read alignment with TopHat and read quantification with Cufflinks using UCSC hg19 transcriptome reference, a total of 189 samples and 23,146 genes were analyzed. A total of 248 genes had zero FPKM values in all samples. After filtering for genes with zero counts in whole samples, noncoding genes, and low variance genes, 16,676 genes were analyzed. Out of these genes, 2,312 genes were differentially expressed between the two groups (FDR corrected *q* < 0.01) (Table 3). There were many overlaps between *t*-test and EdgeR (see supplementary Table 1 in the Supplementary Material available online at  http://dx.doi.org/10.1155/2015/206937). Regression analysis of the DEGs with clinical phenotypes revealed that genes were more associated with FEV_1_ (forced expiratory volume in the first second) and FEV_1_/FVC ratio than with age and smoking history (Figure 2).

### 3.3. Validation

**Figure 3:**
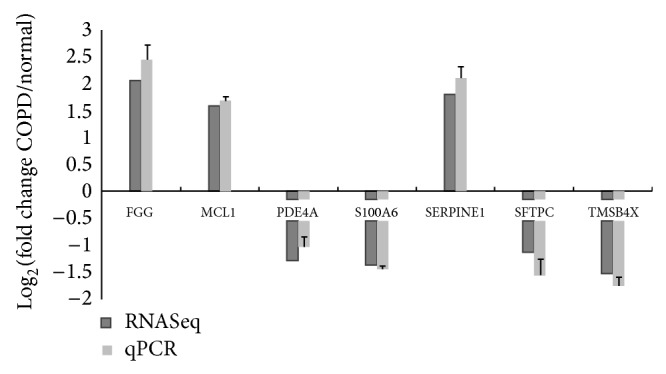
RNA-seq and quantitative real-time PCR results of seven genes. Results of fold change by two methods are shown.

To validate the RNA-seq results, we performed qRT-PCR of the* FGG, MCL1, PDE4A, S100A6, SERPINE1, SFTPC*, and* TMSB4X* genes using the same RNA samples that were used for RNA-seq. We used a subset of 156 samples out of 189 samples for validation. The RNA-seq and qRT-PCR results were in good agreement (Figure 3).

### 3.4. Clustering and Gene Functions

**Figure 4:**
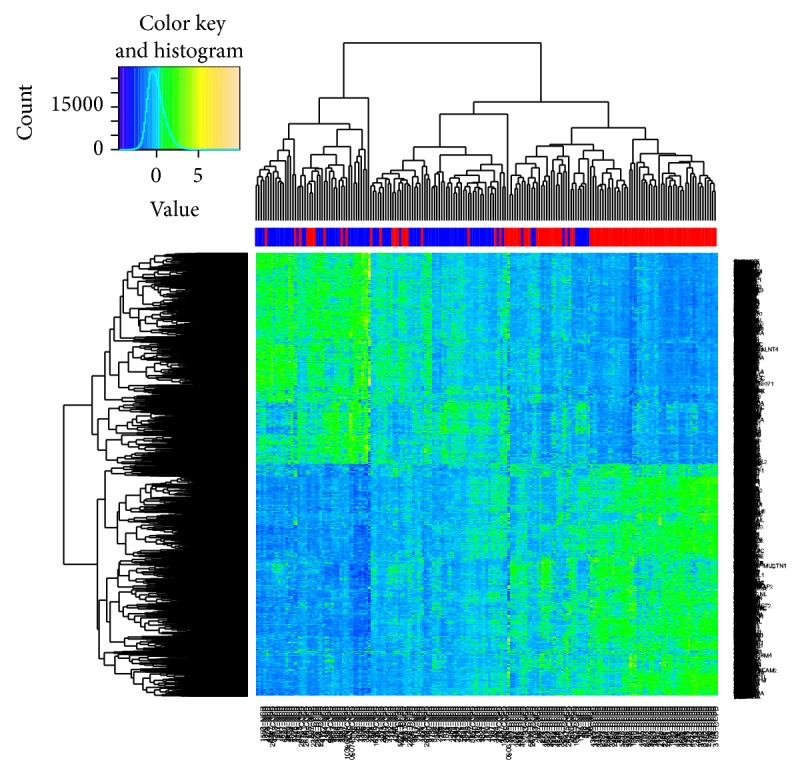
Heat map of RNA-seq results of lung tissues from COPD and control subjects. Color column sidebar indicated status of subjects, where red means COPD subjects and blue means control group.

A heat map shows the genes that were differentially expressed between the two groups (Figure 4). DAVID revealed that the expression of genes involved in protein catabolism, oxidative phosphorylation, and chromatin modification differed most significantly between the two groups (Table 4). Expression of genes encoding proteasome components including* PSMA2, PSMB1, PSMC5*, and* PSMD4* was lower in the COPD group than in the control group. Gene set enrichment analysis revealed that the most significantly downregulated pathways in the COPD group were oxidative phosphorylation and biosynthetic process (FDR *q* < 0.25).

**Figure 5:**
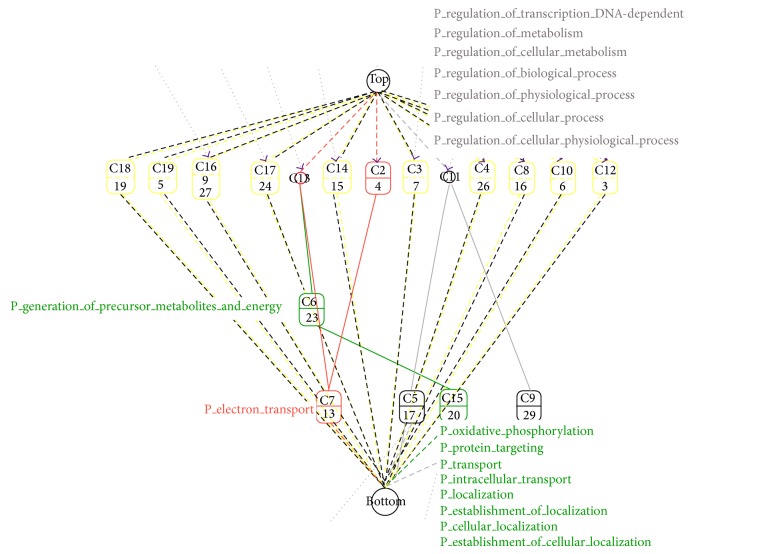
BioLattice analysis of RNA-seq data of lung tissues from COPD and control subjects. Each node indicates significantly enriched GO terms with *P* values < 0.01. Lines indicate significant sharing of genes within two nodes, which indicates similarity of two enriched GO terms with respect to differentially expressed genes.

We used *k*-means clustering to construct 30 groups of coexpressed genes for BioLattice. After matching with hypergeometric distributions to annotate those gene sets to GO terms, concept lattice for coexpressed gene clusters with GO biological process terms was visualized showing that genes related to transcription and oxidative phosphorylation are enriched in the major clusters (Figure 5).

**Figure 6:**
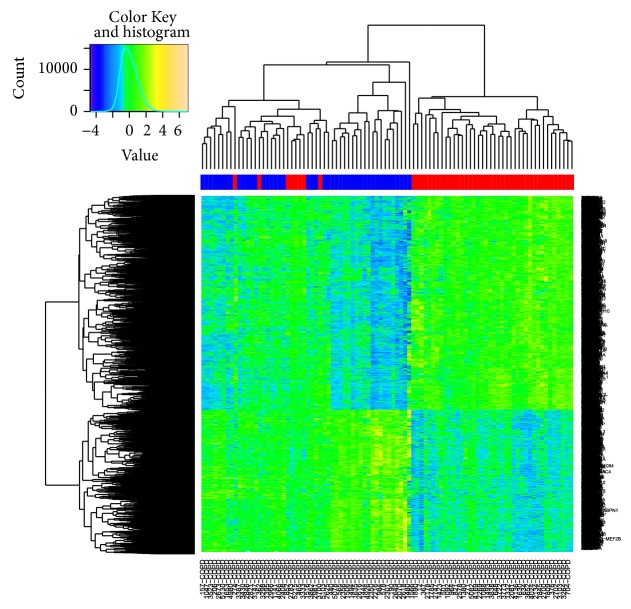
Heat map of RNA-seq results of lung tissues from COPD subjects. Hierarchical clustering of two subgroups in COPD subjects is shown.

In the COPD group, the heat map shows hierarchical clustering of two subgroups using Euclidean distance (Figure 6). Four hundred and four genes that were differentially expressed between these two subgroups in COPD subjects (*q* < 0.05) are enriched in the mitochondrial and steroid biosynthesis pathway. Group 1 showed tendency for lower FEV_1_ and lower DLCO (carbon monoxide diffusing capacity). When comparing each group with the control, DEGs between group 1 and the controls only consisted of 18 genes, and their *P* values were negligible. Meanwhile, DEGs between group 2 and the controls consisted of 4072 genes and their *P* values were even higher than those of DEGs between the COPD group and the controls.

There was no difference in medication history between the two groups.

### 3.5. Isoform and Alternative Splicing

**Figure 7:**
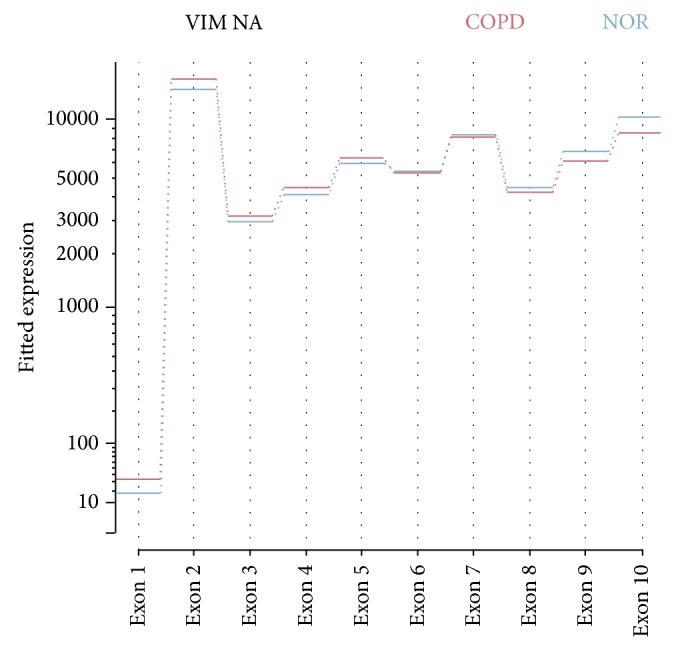
Exon usage of vimentin according to COPD status. COPD subjects showed more exon usage of exon 1 to exon 4.

Isoforms that were differentially expressed between the COPD and control groups were evaluated by Cufflinks. Pathway analysis results of the DEIs were similar to those of the DEGs. However, among the DEIs, 310 were not in the DEG list, which are enriched in genes encoding proteins that function in cell junctions and focal adhesions. The multivariate analysis of transcript splicing program (MATS) revealed that specific alternative splicing events were significantly more common in COPD subjects than in control subjects. Five categories of differentially expressed isoforms were identified by MATS: skipped exon, alternative 5′ splice site, alternative 3′ splice site, mutually exclusive exons, and retained introns. Significantly different events between the COPD group and the control group are shown in the supplementary Table 2 (FDR *q* < 0.01). Intron retention of* HNRNPH1* and* VIM* occurred significantly more in the COPD group than in the control group. Mutually exclusive exons occurred more frequently in the COPD group than in the control group in 78 genes.  Figure 7 shows exon usage of* VIM* between the two groups visualized using DEXSeq [21].

### 3.6. Connectivity Map

A connectivity map [22] was used to identify potential drugs that might reverse the gene expression pattern associated with the pathogenesis of early COPD. Gene expression changes arising from treatment with several drugs were negatively correlated with the expression patterns that differ in the COPD group compared to the control group. Gene expression changes arising from treatment with MG-262 (*P* = 0.00004) and puromycin (*P* < 0.00001) were most significantly negatively correlated.

## 4. Discussion

In this study, we used RNA-seq to identify genes whose expression differs between COPD and control subjects. In total, 2312 genes were identified with FDR *q* < 0.01. We validated a subset of RNA-seq data with qRT-PCR, and the results were in good agreement.

Previous studies have investigated the gene expression profiles of COPD patients using microarray, serial analysis of gene expression, and RNA-seq. Microarray data indicates that* MICAL2* and* NOTCH2* are upregulated in the resected lung tissue of COPD patients [5]; the expression of these genes was higher in the COPD group than in the control group in the present study. The expression of genes encoding ribosomal proteins and* S100A6* was lower in the COPD group than in the control group, and RNA-seq previously indicated that the expression of these genes is reduced in the small airway epithelium of smokers [14]. However, there is little overlap between studies in terms of the genes that are differentially expressed between people with reduced lung function and those with normal lung function [23]. This may be because different methods were used or because different phenotypes were examined. Of interest, 582 differentially expressed genes were not in the Affymetrix microarray (U133a).

Previous studies of the COPD transcriptome reported a high degree of overlap in the biological processes affected. In the current study, the most altered pathway in COPD patients was mitochondrial oxidative phosphorylation. The expression of mitochondrial genes was previously shown to be reduced in the lung tissues of COPD subjects using serial analysis of gene expression [3]. A recent report showed that expression of the mitochondrial membrane protein* PHB1* is downregulated in lung tissue of COPD patients, suggesting that mitochondrial stability is reduced [24]. Another report revealed that mitochondrial mass is reduced in airway epithelial cells exposed to particulate matter, and this defect is also observed in the daughter cells [25]. One possible explanation may be related to the fact that an increase in the CO_2_ level can reduce oxidative phosphorylation [26]. However, considering that our COPD subjects were in relatively early stages of the disease, they are not expected to have a clinically meaningful increase in CO_2_ levels.

In the current study, the protein catabolism pathway was dysregulated in the lung tissues of COPD subjects. Many genes related to the 20S proteasome including* PSMA2, PSMB1, PSMC5, PSMD4*, and* PSMD13*, as well as ubiquitin ligase complex genes including* STUB1, SELS*, and* DERL2*, were downregulated in COPD subjects. The ubiquitination-proteasome pathway is dysregulated in COPD, but the mechanism by which this occurs is not fully understood [27]. The expression of 26S proteasome-associated genes is lower in lung tissues of moderate COPD subjects than in those of the smoker control subjects with normal lung function [3]. The expression and activity of the proteasome are reduced in lung tissues of COPD subjects due to dysregulation of Nrf2 [28]. Impairment of proteasomal activity/expression may be important in the pathogenesis of COPD. Interestingly, the proteasome inhibitor MG-262 was on top of a list of drugs that reverse the gene expression pattern of COPD. In recent experiments in mice, a proteasome inhibitor was suggested to be a therapeutic agent for pulmonary arterial hypertension via inhibition of pulmonary vascular smooth muscle cell proliferation and correction of endothelial dysfunction [29]. Inhibition of proteasome inhibitors can reverse diaphragmatic function in a COPD mouse model [30].

In the current study, chromatin modification genes were upregulated in the COPD group. An epigenetic mechanism is reportedly important in the pathophysiology of COPD [31]. Chromatin modification is an important mechanism in epigenetics. In the current study, the expression of* MLL*, which plays an important role in H3K4 methylation [32], and* CHD*, which is important for chromatin modification and opening of chromatin to allow transcription [33], was increased in COPD subjects. These changes may lead to the upregulation of transcription. The expression of* HDAC10* was decreased in the lung tissue of COPD subjects, while the expression of* HDAC2* was previously reported to be decreased in COPD [34]. Several studies report the mechanism by which the expression of* HDAC2* is reduced in COPD, but further studies are required to explain how the expression of several genes related to chromatin modification is increased in COPD.

In a recent study investigating genes associated with the severity of emphysema, the major pathways affected were inflammation and tissue repair [35]. However, the inflammation pathway was not majorly affected in COPD subjects in the current study, probably because the subjects were at relatively early stages of the disease.

One of the advantages of RNA-seq is that DEIs can be identified. Pathway analysis results of the DEIs were similar to those of the DEGs; however, genes encoding proteins that function in cell junctions and cell migration were found specifically in the DEIs and not in the DEGs. This suggests that specific isoforms of these genes may function in the lung. Alternative splicing of genes is tissue-specific and has important roles in development, physiological responses, and the pathogenesis of diseases [36]. Interestingly, the gene encoding vimentin, which is a structural protein, had more retained introns in the COPD group than in the control group, which may alter the sequence of the protein.

There are several limitations of this study. COPD subjects were older and had more pack years of smoking than the control group. However, regression analysis results showed that most of DEGs had stronger correlation with lung function than age or smoking amount. All subjects were smoking or ex-smoking men. The results of this study may not be applicable to nonsmoking or female COPD subjects. Normal looking tissue adjacent to the lung cancer tissue was used for analysis. Lung tissue consists of many cell types including macrophages, epithelium, and endothelium. Microdissection of lung tissue or single cell sequencing would be required to determine whether the differential expression is altered in all lung cells or only in a specific subset of cells. Finally, it is difficult to determine whether the dysregulated pathways identified in this study are a cause or a consequence of the pathogenesis of COPD. Experiments in which the increase/decrease of the DEGs is reversed and shown to slow disease progression are needed to confirm that these pathways are causally involved in the pathogenesis of COPD.

In conclusion, reduced oxidative phosphorylation, modulation of protein catabolism, and dysregulation of transcription are important molecular features of early stages of COPD. Genes and splicing variants were identified that were differentially expressed between COPD subjects and control subjects. RNA-seq was useful tool to increase understanding of the pathophysiology of COPD.

## Supplementary Material

Summary at a glance: The aim of this study was to identify gene expression profiling of lung tissue using recently developed RNA sequencing technology to define the molecular pathways that are dysregulated in COPD. Oxidative phosphorylation, protein degradation, and chromatin modification were the most dysregulated pathways in the lung tissues of COPD subjects in this study and these findings may have clinical and mechanistic implications in COPD.

## Figures and Tables

**Table 1:** Demographics of COPD subjects and control subjects with normal lung function.

	COPD subjects	Control subjects	*P* value
Male, *n* (%)	98 100.0	91 100.0	
Age, years	67.5 ± 6.4	60.9 ± 9.5	<0.0001
Smoking (py)	48.0 ± 22.0	35.2 ± 17.2	<0.0001
FEV1, %	71.9 ± 13.4	91.0 ± 12.4	<0.0001
FEV1/FVC	57.1 ± 7.8	74.8 ± 4.3	<0.0001
DLCO, %	77.4 ± 13.8	92.8 ± 13.2	<0.0001

COPD: chronic obstructive pulmonary disease; DLCO: diffusing capacity of the lung for CO_2_; pack-years; FEV1: forced expiratory volume in 1 second.

Unless otherwise stated, the mean ± standard deviation is shown.

**Table 2:** Primers for mRNA expression profiling.

Gene symbol	Assay ID	Context sequence
TMSB4X	Hs03407480_gH	GGTGAAGGAAGAAGTGGGGTGGAAG
MCL1	Hs01050896_m1	TAAACAAAGAGGCTGGGATGGGTTT
SFTPC	Hs00161628_m1	AGAGCCCGCCGGACTACTCCGCAGC
S100A6	Hs00170953_m1	CCTCCCTACCGCTCCAAGCCCAGCC
FGG	Hs00241037_m1	TGGAGTTTATTACCAAGGTGGCACT
SERPINE1	Hs01126607_g1	CAACCCCACAGGAACAGTCCTTTTC
PDE4A	Hs01102342_mH	ACCGCATCCAGGTCCTCCGGAACAT

**Table 3:** Top 20 genes differentially expressed between COPD subjects and subjects with normal lung function.

Gene symbol	Gene function	Fold change	*P* value	*q* value	Expression levels
		log_2_⁡(COPD/Normal)			(log_2_⁡(FPKM))
RAD54L2	Androgen receptor-interacting protein	0.70	1.23*E* − 24	2.05*E* − 20	4.13
UBR4	Ubiquitin protein ligase E3 component n-recognin 4	0.46	6.24*E* − 24	1.04*E* − 19	9.71
KPNA6	Karyopherin alpha 6	0.27	2.25*E* − 21	3.75*E* − 17	11.03
VPS28	Vacuolar protein	−0.51	1.11*E* − 20	1.86*E* − 16	60.90
STRA13	Stimulated by retinoic acid	−0.78	2.29*E* − 20	3.82*E* − 16	21.96
SPEN	Spen family transcriptional repressor	0.47	2.68*E* − 20	4.47*E* − 16	9.99
HERC2	HECT and RLD domain containing E3 ubiquitin protein ligase 2	0.42	3.73*E* − 20	6.22*E* − 16	6.05
GTF3C3	General transcription factor IIIC	0.58	6.53*E* − 20	1.09*E* − 15	9.52
TCF20	Transcription factor 20 (AR1)	0.35	7.22*E* − 20	1.20*E* − 15	6.49
MRPL21	Mitochondrial ribosomal protein L21	−0.55	1.91*E* − 19	3.19*E* − 15	22.24
COX6A1	Cytochrome c oxidase subunit VIa polypeptide 1	−0.66	2.17*E* − 19	3.63*E* − 15	237.71
ZZEF1	Zinc finger, ZZ-type with EF-hand domain 1	0.30	2.36*E* − 19	3.94*E* − 15	7.56
STX8	Syntaxin 8	−0.74	3.00*E* − 19	5.00*E* − 15	29.76
UBAP2L	Ubiquitin associated protein 2-like	0.27	3.48*E* − 19	5.80*E* − 15	29.99
TRRAP	Transformation/transcription domain-associated protein	0.40	3.63*E* − 19	6.05*E* − 15	4.96
ENTPD4	Ectonucleoside triphosphate diphosphohydrolase 4	0.47	3.74*E* − 19	6.24*E* − 15	11.88
TRIM56	Tripartite motif containing 56	0.45	4.37*E* − 19	7.30*E* − 15	10.09
NHSL2	NHS-like 2	1.21	4.88*E* − 19	8.14*E* − 15	1.44
SETD5	SET domain containing 5	0.23	5.17*E* − 19	8.62*E* − 15	16.95
PRKAR2A	Protein kinase, cAMP-dependent, regulatory, type II, alpha	0.79	5.39*E* − 19	8.99*E* − 15	8.33

FPKM: fragments per kilobase of exon per million fragments mapped.

**Table 4:** Representative DAVID results of pathway that was differentially expressed between COPD and control groups.

Term	Count of genes involved	Fold enrichment	FDR
GO:0030529~ribonucleoprotein complex	171	2.79	2.76*E* − 35
GO:0070013~intracellular organelle lumen	346	1.63	2.16*E* − 20
GO:0005739~mitochondrion	239	1.85	8.08*E* − 20
GO:0006119~oxidative phosphorylation	36	3.05	1.88*E* − 06
GO:0030163~protein catabolic process	135	1.80	8.98*E* − 09
GO:0006396~RNA processing	125	1.90	1.52*E* − 09
GO:0006351~transcription, DNA-dependent	67	1.90	4.25*E* − 04
GO:0015031~protein transport	138	1.50	1.07*E* − 03
GO:0016568~chromatin modification	61	1.85	4.50*E* − 03
GO:0006511~ubiquitin-dependent protein catabolic process	55	1.89	7.86*E* − 03

## Abbreviations

COPD: Chronic obstructive pulmonary disease
FPKM: Fragments per kilobase per million
DEG: Differentially expressed gene
DEI: Differentially expressed isoform.
