# Supplementary material for: Comprehensive Analysis of Transcriptome Sequencing Data in the Lung Tissues of COPD Subjects
Source: Int J Genomics. 2015 Mar 5;2015:206937. doi: 10.1155/2015/206937 (PMC4365374; doi:10.1155/2015/206937)
Supplement: Supplementary file 1 — Summary at a glance: The aim of this study was to identify gene expression profiling of lung tissue using recently developed RNA sequencing technology to define the molecular pathways that are dysregulated in COPD. Oxidative phosphorylation, protein degradation, and chromatin modification were the most dysregulated pathways in the lung tissues of COPD subjects in this study and these findings may have clinical and mechanistic implications in COPD. [file 206937.f1.zip › CLEAN-RNA-seq_IntJGenomics2014.5_R3.docx]

**Comprehensive Analysis of Transcriptome Sequencing Data**

**in the Lung Tissues of COPD Subjects**

Woo Jin Kim^1*^, Jae Hyun Lim^2*^,

Jae Seung Lee^3^, Sang Do Lee^3^, Ju Han Kim^2†^, Yeon-Mok Oh^3†^

*^1^Department of Internal Medicine and Environmental Health Center, Kangwon National University Hospital, School of Medicine, Kangwon National University, Chuncheon*

*^2^Seoul National University Biomedical Informatics and Systems Biomedical Informatics Research Center, Division of Biomedical Informatics, Seoul National University College of Medicine*

*^3^Department of Pulmonary and Critical Care Medicine, and Clinical Research Center for Chronic Obstructive Airway Diseases, Asan Medical Center, University of Ulsan College of Medicine, Seoul, Korea*

*These authors contributed equally to this work.

†The last two authors acted as equivalent co-senior authors.

**Corresponding Authors:** Yeon-Mok Oh

Department of Pulmonary and Critical Care Medicine, and Clinical Research Center for Chronic Obstructive Airway Diseases, Asan Medical Center, University of Ulsan College of Medicine, Seoul, 138-736, Korea

**Email:** [ymoh55@amc.seoul.kr](mailto:ymoh55@amc.seoul.kr)

**Summary at a glance**: The aim of this study was to identify gene expression profiling of lung tissue using recently developed RNA sequencing technology to define the molecular pathways that are dysregulated in COPD. Oxidative phosphorylation, protein degradation, and chromatin modification were the most dysregulated pathways in the lung tissues of COPD subjects in this study and these findings may have clinical and mechanistic implications in COPD.

Abstract word count: 229 words

Total word count: 2474 words

**Abstract**

**Background and Objectives**: Chronic obstructive pulmonary disease (COPD) is a complex disease characterized by airflow limitation. Although airway inflammation and oxidative stress are known to be important in the pathogenesis of COPD, the mechanism underlying airflow obstruction is not fully understood. Gene expression profiling of lung tissue was performed using recently developed RNA sequencing technology to define the molecular pathways that are dysregulated in COPD.

**Methods:** RNA was isolated from lung tissues obtained from 98 subjects with COPD and 91 control subjects with normal spirometry. The RNA samples were processed with RNA-seq using the HiSeq 2000 system. Gene expression levels were calculated using Cufflinks software. Genes that were significantly differentially expressed between the two groups were identified using Student’s *t*-test. Quantitative real-time PCR was used to validate some of the differentially expressed genes.

**Results:** After filtering for genes with zero counts and non-coding genes, 16,676 genes were evaluated. A total of 2312 genes were differentially expressed between the lung tissues of COPD and control subjects (false discovery rate corrected q < 0.01). The expression of genes related to oxidative phosphorylation and protein catabolism was reduced and genes related to chromatin modification were dysregulated in lung tissues of COPD subjects.

**Conclusions:** Oxidative phosphorylation, protein degradation, and chromatin modification were the most dysregulated pathways in the lung tissues of COPD subjects. These findings may have clinical and mechanistic implications in COPD.

**Keywords:** genetics; gene expression profiling; COPD

**Short title**: transcriptome sequencing data of COPD

**Author Contributions**: This study was conceived by WJK, JHK, and YMO. Data were collected by JSL, YMO, and SDL. Statistical analyses were carried out by WJK, JHL, and JHK. All authors contributed to and approved the final draft of the manuscript.

**Abbreviations:** COPD, Chronic obstructive pulmonary disease; FPKM, fragments per kilobase per million; DEG, differentially expressed gene; DEI, defferentially expressed isoform

**Introduction**

Chronic obstructive pulmonary disease (COPD) is characterized by chronic airflow limitation that is not fully reversible and is highly prevalent worldwide [1]. Although cigarette smoking is a major risk factor for COPD, the mechanism by which inhaled smoke contributes to airflow obstruction is not fully understood. Current theories for this include persistent airway inflammation that is modified by oxidative stress, excess proteinases, autoimmunity, and apoptosis of alveolar cells [2].

Gene expression studies of diseased lungs can generate high-throughput results to shed light on the molecular processes underlying COPD pathogenesis. Whole-genome expression of COPD in humans have been studied using airway epithelium and resected lung tissue in several groups [3-9]. These studies mostly used microarrays to profile gene expression patterns in COPD. Next-generation sequencing technology was recently applied to transcriptomics. RNA-seq technology provides read counts of RNA fragments in each gene [10]. Background and cross-hybridization are not issues in RNA-seq and the technology can quantify both lowly and highly abundant transcripts [11]. In addition, this method can provide information about splice variants, allele-specific expression and fusion transcripts [12]. RNA-seq data of airways was recently published [13, 14]; however, the number of subjects in these studies was relatively small.

We performed gene expression profiling using RNA-seq of lung tissues resected from large numbers of subjects with COPD or with control subjects to better understand the molecular mechanisms responsible for the pathogenesis of COPD.

**Methods**

*Study populations*

Subjects were patients who required resection for lung cancer and who were registered in the Asan Biobank from January 2008 to November 2011. The inclusion criteria was a post-bronchodilator FEV_1_/FVC ratio (ratio of forced expiratory volume in the first second to forced vital capacity)of less than 0.7 for the COPD group, and normal spirometry for the control group in accordance with American Thoracic Society/European Respiratory Society criteria [15]. This study was approved by the institutional review board of Asan Medical Center (2011-0711) and written informed consent was obtained from all patients.

*RNA preparation and sequencing*

Total RNA was isolated from apparently normal fresh frozen lung tissue that was remote from the lung cancer. RNA integrity was assessed using an Agilent Bioanalyzer and RNA purity was assessed using a NanoDrop spectrophotometer.

One µg of total RNA was used to generate cDNA libraries using the TruSeq RNA library kit. The protocol consisted of poly A-selected RNA extraction, RNA fragmentation, reverse transcription using random hexamer primers, and 100 bp paired-end sequencing using the Illumina HiSeq 2000 system. All data have been deposited in the NCBI Gene Expression Omnibus (GEO) public respository and can be accessed through the accession number GSE57148.

*Quality control and data management*

For quality control, read quality was verified using FastQC and read alignment was verified using Picard. Differential gene expression (DEG) analysis was performed using TopHat and Cufflinks software [16]. To estimate expression levels, the RNA-seq reads were mapped to the human genome using TopHat (version 1.4.1) [17], and quantified using Cufflinks software (2.0.0) [18]. Cufflinks software was run with the UCSC hg19 human genome and transcriptome references. The numbers of isoform and gene transcripts were calculated and the relative abundance of transcripts was measured in fragments per kilobase of exon per million fragments mapped (FPKM).

Expression levels were extracted as a FPKM value for each gene of each sample using Cufflinks software. Genes with FPKM values of 0 across all samples were excluded. Filtered data were subject to upper quantile normalization. Statistical significance was determined using Student’s *t*-test. The false discovery rate (FDR) was controlled by adjusting p values using the Benjamini-Hochberg algorithm. The analysis steps used are summarized in Figure 1.

To investigate whether DEGs are related to clinical phenotypes, we performed a linear regression analysis for 5 clinical phenotypes with respect to its gene expression. We considered each clinical phenotype as the responder for regression and each gene expression as the predictor.

*Quantitative real-time PCR (qRT-PCR)*

Several genes whose expression level was found to be related to COPD status by RNA-seq were validated using TaqMan® real-time PCR. The results were normalized to GAPDH Ct values. Primer sequences for the genes of interest are given in Table 2.

*Pathway analysis*

Functional enrichment analysis was performed using Gene Set Enrichment Analysis (version 2.0.8), which combines information from previously defined gene sets obtained from the Molecular Signature Database (version 3.1). Biological gene functional annotation analysis was performed using DAVID (version 6.7) with a list of DEGs. BioLattice (version 1.1) was used to annotate co-expressed gene groups to GO Biological process terms and visualize their relations [19].

*Differential alternative splicing*

To detect differential alternative splicing between the two groups, subjects from each group were evaluated using a multivariate Bayesian algorithm called ‘Multivariate Analysis of Transcript Splicing [20]. Differential alternative splicing, including exon skipping, mutually exclusive exons, alternative 5’ or 3’ splice site usage and intron retention, were investigated. Exon usage of *VIM* between two groups visualized using DEXSeq [21].

*Connectivity map*

A connectivity map [22] was used to identify potential drugs that might reverse the gene expression pattern associated with the pathogenesis of early COPD. The connectivity map is a collection of genome-wide transcriptional expression data from cultured human cells treated with bioactive small molecules. The basic assumption of the connectivity map is that transcriptional perturbation can occurr or be treated by certain drugs that intrigue similar changes.

**Results**

*Demographic characteristics*

The demographic characteristics of 98 subjects with COPD and 91 control subjects are shown in Table 1. All subjects were male and the mean age and the mean pack years of cigarette smoking history was higher in the COPD group than in the control group. As expected, pulmonary function was significantly lower in the COPD group than in the control subjects. Most of COPD subjects were in early stage. In COPD group, 28 subjects took inhaled corticosteroid, 40 subjects took tiotropium, and 22 subjects took short-acting beta-agonist. None in control group took bronchodilator.

*Quality control and DEGs*

The total number of reads produced from each sample was 38,742,474 ± 7,332,014 reads (mean ± standard deviation). The difference in the number of reads between COPD samples and control samples was not statistically significant. After read alignment with TopHat and read quantification with Cufflinks using UCSC hg19 transcriptome reference, a total of 189 samples and 23,146 genes were analyzed. A total of 248 genes had zero FPKM values in all samples. After filtering for genes with zero counts in whole samples, non-coding genes, and low variance genes, 16,676 genes were analyzed. Out of these genes, 2,312 genes were differentially expressed between the two groups (FDR corrected q < 0.01) (Table 3). There were many overlaps between t-test and EdgeR (supplementary table 1). Regression analysis of the DEGs with clinical phenotypes revealed that genes were more associated with FEV_1_ (forced expiratory volume in the first second**)** and FEV_1_/FVC ratio than with age and smoking history (Figure 2).

*Validation*

To validate the RNA-seq results, we performed qRT-PCR of the *FGG, MCL1, PDE4A, S100A6, SERPINE1, SFTPC,* and *TMSB4X* genes using the same RNA samples that were used for RNA-seq. We used a subset of 156 samples out of 189 samples for validation. The RNA-seq and qRT-PCR results were in good agreement (Figure 3).

*Clustering and gene functions*

A heat map shows the genes that were differentially expressed between the two groups (Figure 4). DAVID revealed that the expression of genes involved in protein catabolism, oxidative phosphorylation, and chromatin modification differed most significantly between the two groups (Table 4). Expression of genes encoding proteasome components including *PSMA2, PSMB1, PSMC5,* and *PSMD4* was lower in the COPD group than in the control group. Gene set enrichment analysis revealed that the most significantly down-regulated pathways in the COPD group were oxidative phosphorylation and biosynthetic process (FDR q < 0.25).

We used k-means clustering to construct 30 groups of co-expressed genes for BioLattice. After matching with hypergeometric distributions to annotate those gene sets to GO terms, concept lattice for co-expressed gene clusters with GO biological process terms was visualized showing that genes related to transcription and oxidative phosphorylation are enriched in the major clusters (Figure 5).

In the COPD group, the heat map shows hierarchical clustering of two subgroups using euclidian distance (Figure 6). Four hundred and four genes that were differentially expressed between these two subgroups in COPD subjects (q<0.05) are enriched in the mitochondrial and steroid biosynthesis pathway. Group 1 showed tendency for lower FEV_1_ and lower DLCO (Carbon monoxide diffusing capacity). When comparing each group with the control, DEGs between group 1 and the controls only consisted of 18 genes,and their p values were negligible. Meanwhile, DEGs between group 2 and the controls consisted of 4072 genes and their p values were even higher than that of DEGs between the COPD group and the controls.

There was no difference in medication history between the two groups.

*Isoform and alternative splicing*

Isoforms that were differentially expressed between the COPD and control groups were evaluated by Cufflinks. Pathway analysis results of the DEIs were similar to those of the DEGs. However, among the DEIs, 310 were not in the DEG list, which are enriched in genes encoding proteins that function in cell junctions and focal adhesions. The multivariate analysis of transcript splicing program (MATS) revealed that specific alternative splicing events were significantly more common in COPD subjects than in control subjects. Five categories of differentially expressed isoforms were identified by MATS; skipped exon, alternative 5’splice site, alternative 3’splice site, mutually exclusive exons, and retained introns. Significantly different events between the COPD group and in the control group are shown in the supplementary table 2 (FDR q < 0.01). Intron retention of *HNRNPH1* and *VIM* occurred significantly more in the COPD group than in the control group. Mutually exclusive exons occurred more frequently in the COPD group than in the control group in 78 genes. Figure 7 shows exon usage of *VIM* between the two groups visualized using DEXSeq [21].

*Connectivity map*

A connectivity map [22] was used to identify potential drugs that might reverse the gene expression pattern associated with the pathogenesis of early COPD. Gene expression changes arising from treatment with several drugs were negatively correlated with the expression patterns that differ in the COPD group compared to the control group. Gene expression changes arising from treatment with MG-262 (p = 0.00004) and puromycin (p < 0.00001) were most significantly negatively correlated.

**Discussion**

In this study, we used RNA-seq to identify genes whose expression differs between COPD and control subjects. In total, 2312 genes were identified with FDR q < 0.01. We validated a subset of RNA-seq data with qRT-PCR, and the results were in good agreement.

Previous studies have investigated the gene expression profiles of COPD patients using microarray, serial analysis of gene expression and RNA-seq. Microarray data indicates that *MICAL2* and *NOTCH2* are up-regulated in the resected lung tissue of COPD patients [5]; the expression of these genes was higher in the COPD group than in the control group in the present study. The expression of genes encoding ribosomal proteins and *S100A6* were lower in the COPD group than in the control group, and RNA-seq previously indicated that the expression of these genes is reduced in the small airway epithelium of smokers [14]. However, there is little overlap between studies in terms of the genes that are differentially expressed between people with reduced lung function and those with normal lung function [23]. This may be because different methods were used or because different phenotypes were examined. Of interest, 582 differentially expressed genes were not in the Affymetrix microarray (U133a).

Previous studies of the COPD transcriptome reported a high degree of overlap in the biological processes affected. In the current study, the most altered pathway in COPD patients was mitochondrial oxidative phosphorylation. The expression of mitochondrial genes was previously shown to be reduced in the lung tissues of COPD subjects using serial analysis of gene expression [3]. A recent report showed that expression of the mitochondrial membrane protein *PHB1* is down-regulated in lung tissue of COPD patients, suggesting that mitochondrial stability is reduced [24]. Another report revealed that mitochondrial mass is reduced in airway epithelial cells exposed to particulate matter, and this defect is also observed in the daughter cells [25]. One possible explanation may be related to the fact that an increase in the CO_2_ level can reduce oxidative phosphorylation [26]. However, considering that our COPD subjects were in relatively early stages of the disease, they are not expected to have a clinically meaningful increase in CO_2_ levels.

In the current study, the protein catabolism pathway was dysregulated in the lung tissues of COPD subjects. Many genes related to the 20S proteasome including *PSMA2, PSMB1, PSMC5, PSMD4*, and *PSMD13*, as well as ubiquitin ligase complex genes including *STUB1, SELS*, and *DERL2* were down-regulated in COPD subjects. The ubiquitination-proteasome pathway is dysregulated in COPD, but the mechanism by which this occurs is not fully understood [27]. The expression of 26S proteasome-associated genes is lower in lung tissues of moderate COPD subjects than in those of the smoker control subjects with normal lung function [3]. The expression and activity of the proteasome is reduced in lung tissues of COPD subjects due to dysregulation of Nrf2 [28]. Impairment of proteasomal activity/expression may be important in the pathogenesis of COPD. Interestingly, the proteasome inhibitor MG-262 was top of a list of drugs that reverse the gene expression pattern of COPD. In recent experiments in mice, a proteasome inhibitor was suggested to be a therapeutic agent for pulmonary arterial hypertension via inhibition of pulmonary vascular smooth muscle cell proliferation and correction of endothelial dysfunction [29]. Inhibition of proteasome inhibitors can reverse diaphragmatic function in a COPD mouse model [30].

In the current study, chromatin modification genes were up-regulated in the COPD group. An epigenetic mechanism is reportedly important in the pathophysiology of COPD [31]. Chromatin modification is an important mechanism in epigenetics. In the current study, the expression of *MLL*, which plays an important role in H3K4 methylation [32], and *CHD*, which is important for chromatin modification and opening of chromatin to allow transcription [33], was increased in COPD subjects. These changes may lead to the upregulation of transcription. The expression of *HDAC10* was decreased in the lung tissue of COPD subjects, while the expression of *HDAC2* was previously reported to be decreased in COPD [34]. Several studies report the mechanism by which the expression of *HDAC2* is reduced in COPD, but further studies are required to explain how the expression of several genes related to chromatin modification are increased in COPD.

In a recent study investigating genes associated with the severity of emphysema, the major pathways affected were inflammation and tissue repair [35]. However, the inflammation pathway was not majorly affected in COPD subjects in the current study, probably because the subjects were at relatively early stages of the disease.

One of the advantages of RNA-seq is that DEIs can be identified. Pathway analysis results of the DEIs were similar to those of the DEGs; however, genes encoding proteins that function in cell junctions and cell migration were found specifically in the DEIs and not in the DEGs. This suggests that specific isoforms of these genes may function in the lung. Alternative splicing of genes is tissue-specific and has important roles in development, physiological responses and the pathogenesis of diseases [36]. Interestingly, the gene encoding vimentin, which is a structural protein, had more retained introns in the COPD group than in the control group, which may alter the sequence of the protein.

There are several limitations of this study. COPD subjects were older and had more pack years of smoking than the control group. However, regression analysis results showed that most of DEGs had stronger correlation with lung function than age or smoking amount. All subjects were smoking or ex-smoking men. The results of this study may not be applicable to non-smoking or female COPD subjects. Normal looking tissue adjacent to the lung cancer tissue was used for analysis. Lung tissue consists of many cell types including macrophages, epithelium and endothelium. Microdissection of lung tissue or single cell sequencing would be required to determine whether the differential expression is altered in all lung cells, or only in a specific subset of cells. Finally, it is difficult to determine whether the dysregulated pathways identified in this study are a cause or a consequence of the pathogenesis of COPD. Experiments in which the increase/decrease of the DEGs is reversed and shown to slow disease progression are needed to confirm that these pathways are causally involved in the pathogenesis of COPD.

In conclusion, reduced oxidative phosphorylation, modulation of protein catabolism, and dysregulation of transcription are important molecular features of early stages of COPD. Genes and splicing variants were identified that were differentially expressed between COPD subjects and control subjects. RNA-seq was useful tool to increase understanding of the pathophysiology of COPD.

**Acknowledgements**

This study was supported by the National Project for Personalized Genomic Medicine (A111218-11-GM02), the Korea Healthcare Technology R&D Project, Ministry for Health and Welfare (HI13C1634), and Basic Science Research Program through the National Research Foundation of Korea (NRF) funded by the Ministry of Education, Science and Technology (2012-0000994).

**Conflicts of Interests Statement**

The authors declare that there is no conflict of interests regarding the publication of this article.**References**

[1] Vestbo J, Hurd SS, Agusti AG, et al., "Global Strategy for the Diagnosis, Management, and Prevention of Chronic Obstructive Pulmonary Disease: GOLD Executive Summary," *American Journal of Respiratory and Critical Care Medicine*, vol. 187, no. 4, pp. 347-365, 2013.

[2] Cosio MG, Saetta M, Agusti A, "Immunologic Aspects of Chronic Obstructive Pulmonary Disease," *New England Journal of Medicine*, vol. 360, no. 23, pp. 2445-2454, 2009.

[3] Ning W, Li C-J, Kaminski N, et al., "Comprehensive gene expression profiles reveal pathways related to the pathogenesis of chronic obstructive pulmonary disease," *Proceedings of the National Academy of Sciences of the United States of America*, vol. 101, no. 41, pp. 14895-14900, 2004.

[4] Golpon HA, Coldren CD, Zamora MR, et al., "Emphysema Lung Tissue Gene Expression Profiling," *American Journal of Respiratory Cell and Molecular Biology*, vol. 31, no. 6, pp. 595-600, 2004.

[5] Spira A, Beane J, Pinto-Plata V, et al., "Gene Expression Profiling of Human Lung Tissue from Smokers with Severe Emphysema," *American Journal of Respiratory Cell and Molecular Biology*, vol. 31, no. 6, pp. 601-610, 2004.

[6] Wang I-M, Stepaniants S, Boie Y, et al., "Gene Expression Profiling in Patients with Chronic Obstructive Pulmonary Disease and Lung Cancer," *American Journal of Respiratory and Critical Care Medicine*, vol. 177, no. 4, pp. 402-411, 2008.

[7] Bhattacharya S, Srisuma S, Demeo DL, et al., "Molecular biomarkers for quantitative and discrete COPD phenotypes," *American Journal of Respiratory Cell and Molecular Biology*, vol. 40, no. 3, pp. 359-367, 2009.

[8] Francis S, Larsen J, Pavey S, et al., "Expression profiling identifies genes involved in emphysema severity," *Respiratory Research*, vol. 10, no. 1, pp. 81, 2009.

[9] Steiling K, van den Berge M, Hijazi K, et al., "A Dynamic Bronchial Airway Gene Expression Signature of Chronic Obstructive Pulmonary Disease and Lung Function Impairment," *American Journal of Respiratory and Critical Care Medicine*, vol. 187, no. 9, pp. 933-942, 2013.

[10] Mortazavi A, Williams BA, McCue K, Schaeffer L, Wold B, "Mapping and quantifying mammalian transcriptomes by RNA-Seq," *Nature Methods*, vol. 5, no. 7, pp. 621-628, 2008.

[11] Ozsolak F, Milos PM, "RNA sequencing: advances, challenges and opportunities," *Nature Reviews: Genetics*, vol. 12, no. 2, pp. 87-98, 2011.

[12] Costa V, Aprile M, Esposito R, Ciccodicola A, "RNA-Seq and human complex diseases: recent accomplishments and future perspectives," *European Journal of Human Genetics*, vol. 21, no. pp. 134-142, 2013.

[13] Beane J, Vick J, Schembri F, et al., "Characterizing the Impact of Smoking and Lung Cancer on the Airway Transcriptome Using RNA-Seq," *Cancer Prevention Research*, vol. 4, no. 6, pp. 803-817, 2011.

[14] Hackett N, Butler M, Shaykhiev R, et al., "RNA-Seq quantification of the human small airway epithelium transcriptome," *BMC Genomics*, vol. 13, no. 1, pp. 82, 2012.

[15] Miller MR, Hankinson J, Brusasco V, et al., "Standardisation of spirometry," *European Respiratory Journal*, vol. 26, no. 2, pp. 319-338, 2005.

[16] Trapnell C, Roberts A, Goff L, et al., "Differential gene and transcript expression analysis of RNA-seq experiments with TopHat and Cufflinks," *Nat. Protocols*, vol. 7, no. 3, pp. 562-578, 2012.

[17] Trapnell C, Pachter L, Salzberg SL, "TopHat: discovering splice junctions with RNA-Seq," *Bioinformatics*, vol. 25, no. 9, pp. 1105-1111, 2009.

[18] Trapnell C, Williams BA, Pertea G, et al., "Transcript assembly and quantification by RNA-Seq reveals unannotated transcripts and isoform switching during cell differentiation," *Nat Biotech*, vol. 28, no. 5, pp. 511-515, 2010.

[19] Kim JH, "Chapter 8: Biological Knowledge Assembly and Interpretation," *PLoS Computational Biology*, vol. 8, no. 12, pp. e1002858, 2012.

[20] Shen S, Park JW, Huang J, et al., "MATS: a Bayesian framework for flexible detection of differential alternative splicing from RNA-Seq data," *Nucleic Acids Research*, vol. 40, no. 8, pp. e61, 2012.

[21] Anders S, Reyes A, Huber W, "Detecting differential usage of exons from RNA-seq data," *Genome Research*, vol. 22, no. 10, pp. 2008-2017, 2012.

[22] Lamb J, Crawford ED, Peck D, et al., "The Connectivity Map: Using Gene-Expression Signatures to Connect Small Molecules, Genes, and Disease," *Science*, vol. 313, no. 5795, pp. 1929-1935, 2006.

[23] Zeskind JE, Lenburg ME, Spira A, "Translating the COPD Transcriptome," *Proceedings of the American Thoracic Society*, vol. 5, no. 8, pp. 834-841, 2008.

[24] Soulitzis N, Neofytou E, Psarrou M, et al., "Downregulation of lung mitochondrial prohibitin in COPD," *Respiratory Medicine*, vol. 106, no. 7, pp. 954-961, 2012.

[25] Di Pietro A, Visalli G, Baluce B, et al., "Multigenerational mitochondrial alterations in pneumocytes exposed to oil fly ash metals," *International Journal of Hygiene and Environmental Health*, vol. 214, no. 2, pp. 138-144, 2011.

[26] Vohwinkel CU, Lecuona E, Sun H, et al., "Elevated CO2 Levels Cause Mitochondrial Dysfunction and Impair Cell Proliferation," *Journal of Biological Chemistry*, vol. 286, no. 43, pp. 37067-37076, 2011.

[27] Meiners S, Eickelberg O, "What shall we do with the damaged proteins in lung disease? Ask the proteasome!," *European Respiratory Journal*, vol. 40, no. 5, pp. 1260-1268, 2012.

[28] Malhotra D, Thimmulappa R, Vij N, et al., "Heightened Endoplasmic Reticulum Stress in the Lungs of Patients with Chronic Obstructive Pulmonary Disease," *American Journal of Respiratory and Critical Care Medicine*, vol. 180, no. 12, pp. 1196-1207, 2009.

[29] Kim S-Y, Lee J-H, Huh JW, et al., "Bortezomib Alleviates Experimental Pulmonary Arterial Hypertension," *American Journal of Respiratory Cell and Molecular Biology*, vol. 47, no. 5, pp. 698-708, 2012.

[30] van Hees H, Ottenheijm C, Ennen L, Linkels M, Dekhuijzen R, Heunks L, "Proteasome inhibition improves diaphragm function in an animal model for COPD," *American Journal of Physiology - Lung Cellular and Molecular Physiology*, vol. 301, no. 1, pp. L110-L116, 2011.

[31] Yang IV, Schwartz DA, "Epigenetic Control of Gene Expression in the Lung," *American Journal of Respiratory and Critical Care Medicine*, vol. 183, no. 10, pp. 1295-1301, 2011.

[32] Wang P, Lin C, Smith ER, et al., "Global Analysis of H3K4 Methylation Defines MLL Family Member Targets and Points to a Role for MLL1-Mediated H3K4 Methylation in the Regulation of Transcriptional Initiation by RNA Polymerase II," *Molecular and Cellular Biology*, vol. 29, no. 22, pp. 6074-6085, 2009.

[33] Murawska M, Brehm A, "CHD chromatin remodelers and the transcription cycle," *Transcription*, vol. 2, no. 6, pp. 244-253, 2011.

[34] Ito K, Ito M, Elliott WM, et al., "Decreased Histone Deacetylase Activity in Chronic Obstructive Pulmonary Disease," *New England Journal of Medicine*, vol. 352, no. 19, pp. 1967-1976, 2005.

[35] Campbell JD, McDonough JE, Zeskind JE, et al., "A gene expression signature of emphysema-related lung destruction and its reversal by the tripeptide GHK," *Genome Medicine*, vol. 4, no. 8, pp. 67, 2012.

[36] Kalsotra A, Cooper TA, "Functional consequences of developmentally regulated alternative splicing," *Nature Reviews: Genetics*, vol. 12, no. 10, pp. 715-729, 2011.

Table 1: Demographics of COPD subjects and control subjects with normal lung function.

|  | COPD subjects | Control subjects | p value |
| --- | --- | --- | --- |
| Male, n (%) | 98 (100.0) | 91 (100.0) |  |
| Age, years | 67.5±6.4 | 60.9±9.5 | <0.0001 |
| Smoking (py) | 48.0±22.0 | 35.2±17.2 | <0.0001 |
| FEV1, % | 71.9±13.4 | 91.0±12.4 | <0.0001 |
| FEV1/FVC | 57.1±7.8 | 74.8±4.3 | <0.0001 |
| DLCO, % | 77.4±13.8 | 92.8±13.2 | <0.0001 |

COPD, chronic obstructive pulmonary disease; DLCO, diffusing capacity of the lung for CO_2_; pack-years; FEV1, forced expiratory volume in 1 second

Unless otherwise stated, the mean ± standard deviation is shown.

Table 2: Primers for mRNA expression profiling.

| Gene Symbol | Assay ID | Context Sequence |
| --- | --- | --- |
| TMSB4X | Hs03407480_gH | GGTGAAGGAAGAAGTGGGGTGGAAG |
| MCL1 | Hs01050896_m1 | TAAACAAAGAGGCTGGGATGGGTTT |
| SFTPC | Hs00161628_m1 | AGAGCCCGCCGGACTACTCCGCAGC |
| S100A6 | Hs00170953_m1 | CCTCCCTACCGCTCCAAGCCCAGCC |
| FGG | Hs00241037_m1 | TGGAGTTTATTACCAAGGTGGCACT |
| SERPINE1 | Hs01126607_g1 | CAACCCCACAGGAACAGTCCTTTTC |
| PDE4A | Hs01102342_mH | ACCGCATCCAGGTCCTCCGGAACAT |

Table 3: Top 20 genes differentially expressed between COPD subjects and subjects with

normal lung function.

| Gene Symbol | Gene Function | Fold Change  Log_2_(COPD/Normal) | p value | q value | Expression levels (log_2_(FPKM)) |
| --- | --- | --- | --- | --- | --- |
| RAD54L2 | Androgen Receptor-Interacting Protein | 0.70 | 1.23E-24 | 2.05E-20 | 4.13 |
| UBR4 | ubiquitin protein ligase E3 component n-recognin 4 | 0.46 | 6.24E-24 | 1.04E-19 | 9.71 |
| KPNA6 | karyopherin alpha 6 | 0.27 | 2.25E-21 | 3.75E-17 | 11.03 |
| VPS28 | vacuolar protein | -0.51 | 1.11E-20 | 1.86E-16 | 60.90 |
| STRA13 | stimulated by retinoic acid | -0.78 | 2.29E-20 | 3.82E-16 | 21.96 |
| SPEN | spen family transcriptional repressor | 0.47 | 2.68E-20 | 4.47E-16 | 9.99 |
| HERC2 | HECT and RLD domain containing E3 ubiquitin protein ligase 2 | 0.42 | 3.73E-20 | 6.22E-16 | 6.05 |
| GTF3C3 | general transcription factor IIIC | 0.58 | 6.53E-20 | 1.09E-15 | 9.52 |
| TCF20 | transcription factor 20 (AR1) | 0.35 | 7.22E-20 | 1.20E-15 | 6.49 |
| MRPL21 | mitochondrial ribosomal protein L21 | -0.55 | 1.91E-19 | 3.19E-15 | 22.24 |
| COX6A1 | cytochrome c oxidase subunit VIa polypeptide 1 | -0.66 | 2.17E-19 | 3.63E-15 | 237.71 |
| ZZEF1 | zinc finger, ZZ-type with EF-hand domain 1 | 0.30 | 2.36E-19 | 3.94E-15 | 7.56 |
| STX8 | syntaxin 8 | -0.74 | 3.00E-19 | 5.00E-15 | 29.76 |
| UBAP2L | ubiquitin associated protein 2-like | 0.27 | 3.48E-19 | 5.80E-15 | 29.99 |
| TRRAP | transformation/transcription domain-associated protein | 0.40 | 3.63E-19 | 6.05E-15 | 4.96 |
| ENTPD4 | ectonucleoside triphosphate diphosphohydrolase 4 | 0.47 | 3.74E-19 | 6.24E-15 | 11.88 |
| TRIM56 | tripartite motif containing 56 | 0.45 | 4.37E-19 | 7.30E-15 | 10.09 |
| NHSL2 | NHS-like 2 | 1.21 | 4.88E-19 | 8.14E-15 | 1.44 |
| SETD5 | SET domain containing 5 | 0.23 | 5.17E-19 | 8.62E-15 | 16.95 |
| PRKAR2A | protein kinase, cAMP-dependent, regulatory, type II, alpha | 0.79 | 5.39E-19 | 8.99E-15 | 8.33 |

FPKM= fragments per kilobase of exon per million fragments mapped

Table 4. Representative DAVID results of pathway that was differentially expressed between COPD and control groups

| Term | Count of genes involved | Fold Enrichment | FDR |  |
| --- | --- | --- | --- | --- |
| GO:0030529~ribonucleoprotein complex | 171 | 2.79 | 2.76E-35 |  |
| GO:0070013~intracellular organelle lumen | 346 | 1.63 | 2.16E-20 |  |
| GO:0005739~mitochondrion | 239 | 1.85 | 8.08E-20 |  |
| GO:0006119~oxidative phosphorylation | 36 | 3.05 | 1.88E-06 |  |
| GO:0030163~protein catabolic process | 135 | 1.80 | 8.98E-09 |  |
| GO:0006396~RNA processing | 125 | 1.90 | 1.52E-09 |  |
| GO:0006351~transcription, DNA-dependent | 67 | 1.90 | 4.25E-04 |  |
| GO:0015031~protein transport | 138 | 1.50 | 1.07E-03 |  |
| GO:0016568~chromatin modification | 61 | 1.85 | 4.50E-03 |  |
| GO:0006511~ubiquitin-dependent protein catabolic process | 55 | 1.89 | 7.86E-03 |  |

**Figure legends**

Figure 1. Schematic overview of the transcript analysis of RNA-seq experiment. Briefly, we used tophat to align raw fastq files and used cufflinks to read annotation and quantification. FastQC was used to check read quality.

Figure 2. Regression analysis of the differentially expressed genes with clinical phenotypes. Genes were more associated with FEV_1_ (forced expiratory volume in 1 second**)** and FEV_1_/FVC ratio (ratio of forced expiratory volume in 1 second to forced vital capacity) than with age and smoking history.

Figure 3. RNA-seq and quantitative real-time PCR results of seven genes. Results of fold change by two methods are shown.

Figure 4. Heat map of RNA-seq results of lung tissues from COPD and control subjects. Color column sidebar indicated status of subjects, where red means COPD subjects and blue means control group.

Figure 5. BioLattice analysis of RNA-seq data of lung tissues from COPD and control subjects. Each nodes indicate significantly enriched GO terms with p values < 0.01. Lines indicate significant sharing of genes within two nodes, which indicates similarity of two enriched GO terms with respect to differentially expressed genes.

Figure 6. Heat map of RNA-seq results of lung tissues from COPD subjects. Hierarchical clustering of two subgroups in COPD subjects is shown.

Figure 7. Exon usage of vimentin according to COPD status. COPD subjects showed more exon usage of exon 1 to exon 4.

Supplementary Table 1. EdgeR results of 1420 genes which were significantly differentially expressed between COPD subjects and subjects with normal lung function (FDR < 0.05).
