# Supplementary material for: Comprehensive Analysis of Transcriptome Sequencing Data in the Lung Tissues of COPD Subjects
Source: Int J Genomics. 2015 Mar 5;2015:206937. doi: 10.1155/2015/206937 (PMC4365374; doi:10.1155/2015/206937)
Supplement: Supplementary file 1 — Summary at a glance: The aim of this study was to identify gene expression profiling of lung tissue using recently developed RNA sequencing technology to define the molecular pathways that are dysregulated in COPD. Oxidative phosphorylation, protein degradation, and chromatin modification were the most dysregulated pathways in the lung tissues of COPD subjects in this study and these findings may have clinical and mechanistic implications in COPD. [file 206937.f1.zip › response to rereviewCOPD.docx]

Re-review of “Comprehensive Analysis of Transcriptome Sequencing Data in the Lung Tissues of COPD Subjects”

1. There are now two Supplementary Table 1’s (edgeR results and alternative spliced genes). One should be renamed and referenced properly in the text.

**Response 1: We have renamed alternative spliced genes as supplementary Table 2 (page 11 line 8).**

1. The edgeR supplementary table 1 is still not mentioned in the text.

**Response 2: We mentioned edgeR supplementary table 1 in** **page 9, line 12.**

1. Table 4 is still not mentioned in the text.

**Response 3: We have mentioned Table 4 in page 10 line 3.**

1. There are no supplementary table captions (this is needed at least for the edger results supplementary table).

**Response 4: We added table caption for edger results in the page 29.**
