# Supplementary material for: Comprehensive Analysis of Transcriptome Sequencing Data in the Lung Tissues of COPD Subjects
Source: Int J Genomics. 2015 Mar 5;2015:206937. doi: 10.1155/2015/206937 (PMC4365374; doi:10.1155/2015/206937)
Supplement: Supplementary file 1 — Summary at a glance: The aim of this study was to identify gene expression profiling of lung tissue using recently developed RNA sequencing technology to define the molecular pathways that are dysregulated in COPD. Oxidative phosphorylation, protein degradation, and chromatin modification were the most dysregulated pathways in the lung tissues of COPD subjects in this study and these findings may have clinical and mechanistic implications in COPD. [file 206937.f1.zip › Supplementary Table 2.docx]

Supplementary Table 2**.** 143 genes alternatively spliced between COPD subjects and subjects with normal lung functions.

| Types | Gene Symbol | p-value |
| --- | --- | --- |
| Alternative 3 splicing site | MED24 | 2.14E-02 |
|  | TNK2 | 4.49E-02 |
|  | MARK2 | 2.09E-04 |
|  | DNAJC4 | 3.27E-02 |
|  | KRTCAP2 | 4.75E-02 |
| Alternative 5 splicing site | UBC | 5.65E-03 |
|  | SKA2 | 4.62E-03 |
|  | ADARB1 | 8.51E-03 |
| Mutual exclusive exons | THRAP3 | 1.32E-03 |
|  | CASP8 | 4.55E-02 |
|  | PMS1 | 3.72E-03 |
|  | WBSCR22 | 4.16E-02 |
|  | ADD1 | 4.39E-02 |
|  | MARCH2 | 3.78E-02 |
|  | RBX1 | 3.53E-02 |
|  | CTSC | 1.32E-04 |
|  | AIP | 1.49E-02 |
|  | BCL6 | 9.31E-03 |
|  | PLEKHM2 | 1.04E-02 |
|  | CD48 | 4.03E-03 |
|  | PHF3 | 1.17E-02 |
|  | NBPF14 | 4.04E-03 |
|  | NBPF15 | 4.04E-03 |
|  | NBPF10 | 4.04E-03 |
|  | NBPF12 | 4,04E-03 |
|  | NBPF1 | 4.04E-03 |
|  | NBPF11 | 4.04E-03 |
|  | AE01 | 4.04E-03 |
|  | LOC200030 | 4.04E-03 |
|  | KIAA1245 | 4.04E-03 |
|  | NBPF9 | 4.04E-03 |
|  | NBPF8 | 4.04E-03 |
|  | PFDN5 | 4.54E-02 |
|  | COX6B1 | 1.07E-03 |
|  | RAP1B | 3.82E-02 |
|  | MPDU1 | 2.63E-02 |
|  | NAPSA | 1.19E-03 |
|  | RRP8 | 3.38E-02 |
|  | DPH2 | 3.82E-02 |
|  | C15ORF24 | 1.33E-02 |
|  | LDHA | 2.56E-02 |
|  | IFI44 | 1.41E-04 |
|  | GTF2A2 | 3.39E-02 |
|  | HAX1 | 4.72E-02 |
|  | EPHX1 | 1.27E-02 |
|  | ZMYM4 | 3.71E-02 |
|  | TAGLN | 3.06E-02 |
|  | CCT5 | 2.92E-03 |
|  | TXNDC11 | 3.81E-02 |
|  | DAB2 | 5.73E-04 |
|  | ARPC2 | 1.33E-02 |
|  | RPN1 | 2.57E-02 |
|  | FTHL3 | 9.88E-04 |
|  | FTHL16 | 9.88E-04 |
|  | FTHL20 | 9.88E-04 |
|  | FTH1 | 9.88E-04 |
|  | SFTPC | 5.41E-04 |
|  | SFTPB | 2.55E-02 |
|  | HNRNPH1 | 4.18E-02 |
|  | PARP14 | 4.96E-02 |
|  | TCN2 | 3.38E-02 |
|  | MUC1 | 2.03E-03 |
|  | PTMAP4 | 2.34E-02 |
|  | LOC728026 | 2.34E-02 |
|  | LOC441454 | 2.34E-02 |
|  | PTMA | 2.34E-02 |
|  | TCEA1P2 | 1.23E-02 |
|  | TCEA1 | 1.23E-02 |
|  | GRK5 | 1.25E-02 |
|  | AGER | 5.03E-03 |
|  | MICA | 3.45E-02 |
|  | SIPA1 | 1.58E-04 |
| Retained intron | CLK1 | 3.96E-02 |
|  | VIM | 2.14E-05 |
|  | SEC31B | 3.10E-02 |
|  | DDX24 | 4.41E-02 |
|  | APEX1 | 4.44E-02 |
|  | SLC29A1 | 6.94E-03 |
|  | ARAP3 | 2.50E-02 |
|  | RBM39 | 4.27E-02 |
|  | LOC643167 | 4.27E-02 |
|  | P2RX4 | 2.36E-02 |
|  | MYO1G | 1.34E-02 |
|  | CREBZF | 2.82E-02 |
|  | SELENBP1 | 3.19E-02 |
|  | EIF3M | 9.25E-03 |
|  | UBC | 3.46E-02 |
|  | OXA1L | 4.57E-02 |
|  | U2AF1 | 1.17E-08 |
|  | LMNA | 1.48E-03 |
|  | TTC14 | 5.23E-05 |
|  | MARS | 2.02E-03 |
|  | HNRNPH1 | 2.35E-02 |
|  | MT1L | 2.96E-02 |
|  | MT1E | 2.96E-02 |
|  | MT1JP | 2.96E-02 |
|  | MT1P3 | 2.96E-02 |
|  | AHSA2 | 4.16E-02 |
|  | PLK3 | 7.30E-03 |
|  | ARL6IP4 | 1.66E-02 |
|  | LOC284685 | 9.94E-04 |
|  | EWSR1 | 9.94E-04 |
|  | PTMAP4 | 2.65E-03 |
|  | LOC728026 | 2.65E-03 |
|  | LOC441454 | 2.65E-03 |
|  | PTMA | 2.65E-03 |
|  | ADARB1 | 3.16E-02 |
|  | AP1G2 | 1.04E-02 |
| Skipped Exon | LAMP2 | 2.91E-04 |
|  | ZZZ3 | 1.23E-02 |
|  | CELF2 | 1.33E-03 |
|  | PLD3 | 3.08E-02 |
|  | NAMPT | 4.70E-02 |
|  | TCERG1 | 2.65E-04 |
|  | ERRFI1 | 3.97E-03 |
|  | SFRS11 | 1.40E-02 |
|  | TNFAIP3 | 1.33E-15 |
|  | SLC25A16 | 4.38E-03 |
|  | PFDN5 | 3.74E-02 |
|  | PRMT7 | 1.63E-02 |
|  | NIP7 | 2.46E-03 |
|  | NASP | 4.44E-04 |
|  | LDHA | 2.22E-02 |
|  | IFI44 | 2.19E-02 |
|  | LNX2 | 1.11E-03 |
|  | ITGAX | 4.94E-02 |
|  | SRP9L1 | 1.23E-03 |
|  | SRP9 | 1.23E-03 |
|  | GUK1 | 3.95E-02 |
|  | C5ORF28 | 3.98E-02 |
|  | MBNL1 | 7.94E-03 |
|  | AHCTF1P1 | 1.87E-02 |
|  | AHCTF1 | 1.87E-02 |
|  | MS4A7 | 3.54E-02 |
|  | RNF181 | 3.70E-02 |
|  | TOR1AIP2 | 7.48E-03 |
|  | GUSB | 8.58E-03 |
|  | NUDT9 | 1.20E-02 |
|  | SDR16C5 | 1.21E-02 |
|  | PARP14 | 1.70E-02 |
|  | SKA2 | 1.11E-02 |
|  | FAM114A1 | 3.93E-03 |
|  | STK19 | 3.30E-02 |
